# Susceptibility of *Aeromonas salmonicida* subsp. *salmonicida* bacteria from French farmed trout to antibiotics commonly used in fish farming, and attempt to set epidemiological cut-off values

**DOI:** 10.3389/fmicb.2025.1532748

**Published:** 2025-03-11

**Authors:** Antoine Rostang, Florine Bachelet, Catherine Fournel, Titouan Carabin, Nora Navarro-Gonzalez, Ségolène Calvez

**Affiliations:** Oniris, INRAE, BIOEPAR, Nantes, France

**Keywords:** MIC, *Aeromonas*, trout, antibiotics, furunculosis, COWT, ECV, epidemiologic

## Abstract

*Aeromonas salmonicida* subsp. *salmonicida* (ASS) is a bacterium that can cause opportunistic infections in humans and animals. In farmed rainbow trout it causes furunculosis, with more frequent outbreaks when water temperatures are higher, an additional consequence of global warming. When infections occur, antibiotics are sometimes required. However, data on ASS susceptibility is limited. The aim of this study was to determine the distribution of Minimum Inhibitory Concentrations (MICs) of eight antibiotics commonly used in fish veterinary medicine in a population of pathogenic ASS, and to calculate provisional epidemiological cut-off values (called CO_*Epid*_). To date, only four epidemiological cut-off values (ECV) have been established by CLSI, and none by EUCAST. In this study, 406 bacterial strains of ASS were collected exclusively from diseased French farmed trout over a 10-year period (2012–2021). A combination of PCR, MALDI-TOF and specific characteristics of the bacterial culture was used to identify each isolate to species level. All MIC data were obtained by the broth dilution method according to CLSI recommendations. Our CO_*Epid*_ meets the CLSI defined ECV for florfenicol (4 mg/L) and oxytetracycline (1 mg/L). In the absence of a defined ECV, we proposed a CO_*Epid*_ of 1 mg/L for doxycycline. For sulfadiazine alone, all strains tested were non-wild-type (NWT) with very high MICs. The CO_*Epid*_ was calculated as 4.8/0.25 mg/L for sulfadiazine + trimethoprim (one two-fold dilution difference from the ECV established by CLSI for ormetoprim + sulfadimethoxine). For quinolones, CO_*Epid*_ were 4 mg/L, 4 mg/L and 0.5 mg/L for oxolinic acid, flumequine and enrofloxacin, respectively, with a cross-resistance mechanism. This CO_*Epid*_ for oxolinic acid far exceeds the CLSI defined ECV (0.125 mg/L). A total of 12 strains (3%) were classified as NWT for all antibiotics tested. Over the period studied, the proportion of bacteria susceptible to the different molecules remained stable, except for the tetracycline family. These data will be available to establish internationally agreed epidemiological cut-off values, which are lacking for some antibiotics. These cut-offs are essential to assess and monitor the emergence of bacterial populations with resistance traits, and to establish clinical breakpoints for better use of antimicrobials in fish.

## 1 Introduction

Fish consumption has been increasing worldwide for many years. Given the limited availability of fish products from fisheries, aquaculture appears to be a promising solution to this growing demand. The salmonid farming industry is entirely in line with this trend, with a growth in production volumes. This is especially the case for rainbow trout (*Oncorhynchus mykiss*), which is the most commonly produced freshwater salmonid, with Europe as the main production area. Nevertheless, the aquatic environment is at the crossroads of human, animal and environmental health. Indeed, the aquatic environment may be an ideal setting for the selection and the spread of antibiotic resistance, due to anthropogenic activities (such as wastewater treatment plants or animal farms) that mix bacteria of different origins with residues of antimicrobials or biocides (Baron et al., 2017; Jones et al., 2023). Moreover, most aquaculture production takes place in an open environment, with no biological or chemical water treatment either before or after the farm (*i.e.*, flow-through systems). To encourage the most appropriate use of antibiotics, which are sometimes necessary on farms, and to assess the spread of antimicrobial resistance through water, it is crucial to better characterize the susceptibility of fish pathogenic bacteria to antibiotics.

*Aeromonas* is an aquatic bacterial genus that can be isolated from water samples and aquatic animals in both pristine and polluted environments (Baron et al., 2017). The 36 species contained in this genus are Gram-negative facultative anaerobic bacilli (Dallaire-Dufresne et al., 2014; Fernández-Bravo and Figueras, 2020). Within this genus, the phylogeny is still the subject of debate, especially given the high degree of genetic similarity between most species (Menanteau-Ledouble et al., 2016). Currently, the *Aeromonas* genus is considered not only to be an important fish pathogen, but also to be associated with various infectious complications in both immunocompetent and immunocompromised humans (Janda and Abbott, 2010; Fernández-Bravo and Figueras, 2020). In addition, transformation, *i.e*., the ability of bacteria to laterally transfer genes associated with toxin production, biofilm formation or antibiotic resistance, is a natural property of *Aeromonas* isolates that can accelerate the spread of these genes at the bacterial population level (Huddleston et al., 2013).

*Aeromonas salmonicida* is a non-motile psychrophilic species made up of five subspecies (*salmonicida, achromogenes, masoucida, pectinolytica, and smithia*) (Dallaire-Dufresne et al., 2014). *Aeromonas salmonicida* subsp *salmonicida* (ASS) is the causative agent of furunculosis, a systemic disease of salmonids characterized by high mortality and morbidity in wild and farmed fish in both fresh and salt water. The other four subspecies, as well as all unclassifiable isolates of *Aeromonas salmonicida*, are generally considered to be atypical *A. salmonicida* (Dallaire-Dufresne et al., 2014). Furunculosis is an ubiquitous disease that is widespread throughout the world. This bacterium, which sits at the intersection of all components of One Health, from economic losses in aquaculture to antibiotic-resistant bacteria selected from the environment, is able to rapidly colonize and cause opportunistic infections in humans and animals (Lamy et al., 2022). In rainbow trout, this disease tends to occur in the summer months, when water temperatures are higher. Global warming is therefore a concern for the sustainability of this food production, along with current changes in eating habits across Europe. The shift toward a preference for smoked trout filets is leading to an adaptation of the trout farming industry, as a larger trout size is required for filet processing and therefore the farming period needs to be extended. Whereas rainbow trout were traditionally reared for around 12 months to a final weight of 250 g, current practices tend to extend this to >24 months with the aim of achieving a final weight of 2–3 kg. The economic impact of diseases such as furunculosis is greatly increased by these concurrent environmental and farming changes, as farmers suffer greater losses when morbidity and mortality occur in older, larger fish. The current management of this disease relies on vaccines and antibiotics, the most effective solutions. Unfortunately, vaccines have several limitations: technical difficulties during the primary vaccination (ideally, two injections should be given, but this is not always the case), decreased immune protection at low water temperature or over time, side effects following vaccination, etc (Moreau et al., 2024). When furunculosis occurs, the veterinarian uses antibiotics, almost exclusively distributed *via* medicated feed. In France, the antibiotics used as first-line treatment for furonculosis in trout farms are mainly florfenicol (38% of veterinary drugs prescribed) and the combination of sulfadiazine + trimethoprim (31% of veterinary drugs prescribed), and to a lesser degree oxolinic acid, flumequine, oxytetracycline, amoxicillin or enrofloxacin (Carabin, 2022). Oxolinic acid, flumequine, and oxytetracycline are the only 3 drugs approved for use in fish in France, the others could be used off label *via* prescription cascade.

The Clinical and Laboratory Standards Institute (CLSI) provides internationally harmonized consensus epidemiological cut-off values (ECVs) for susceptibility data to a range of antimicrobial agents for several species of bacteria isolated from aquatic animals (CLSI, 2020b), data obtained using the method described in supplement VET03 (CLSI, 2020a). The epidemiological cut-off value is used to distinguish a wild-type (WT) bacterial subpopulation from a non-wild-type (NWT) subpopulation. The number of laboratories and the number of observations determine the predictive ability of an epidemiological cut-off value. To date, only 4 ECVs for *Aeromonas salmonicida* have been established by CLSI for the following antibiotics: florfenicol, ormetoprim + sulfadimethoxine, oxytetracycline, oxolinic acid (CLSI, 2020b). Our study provides new MIC data, obtained using the standardized microdilution method described in the VET03 supplement, which requires the use of cation-adjusted Mueller-Hinton (MH) broth and incubation at 22°C for 44–48 h (CLSI, 2020a), from a specific subpopulation of *Aeromonas salmonicida* (the subsp. *salmonicida*) isolated exclusively from diseased trout in French fish farms. This study will allow the specific epidemiology of this bacterium in French livestock farms to be studied for antibiotics for which an ECV exists. Compliance with internationally accepted procedures allows data from different studies to be compared over time, and ultimately aggregated to refine ECVs (Smith et al., 2024). In the absence of defined ECV for a given antibiotic, this study proposes provisional epidemiological cut-off values [following internationally accepted methods developed by Kronvall (2010) and Turnidge et al. (2006)] and compares them with the data available in the current literature (in particular other provisional cut-off values). These cut-off values are crucial for assessing and monitoring the emergence of bacterial populations with resistance traits or for establishing clinical breakpoints for better use of antimicrobials in fish.

## 2 Materials and methods

### 2.1 Bacterial isolates

A total of 415 bacterial strains were obtained from the retrospective collections (2012–2021) of two partner laboratories specializing in the diagnostics of fish diseases, GDSAA in the New Aquitaine region (south-west France) and LABOCEA in the Brittany region (north-west France). Only bacteria identified as *Aeromonas salmonicida salmonicida (ASS)* by the partner laboratories were collected. All strains were isolated from samples obtained from fish exhibiting clinical signs of furunculosis, by fish farming veterinarians in various regions of France. This isolates were identified at the species level in the partner laboratories using bacterial culture characteristics [morphology and growth rate of colonies in Petri dishes, dissociation of the culture with a mixture of small and large colonies from a perfectly isolated clone, specific brown pigmentation of ASS in TSA (Tryptic Soy Agar) medium], and a MALDI-TOF (Bruker mass spectrometer) score above 1.9. The geographical origin of our strains reflects the location of the partner laboratories in the Brittany and New Aquitaine regions: 58% of our strains originated from the Brittany region and 24% from the New Aquitaine region.

The strains, stored at −80°C, were streaked on TSA at the two partner laboratories and transported to our laboratory at room temperature. To ensure the purity of the strains, they were streaked again on receipt. Identification of isolates was then confirmed in our laboratory by the PCR method of Byers et al. (2002) and by culture characteristics, in particular brown pigmentation at late stationary phase in TSA medium. All confirmed isolates were then stored in Tryptic Soy Broth (TSB) with 40% glycerol at −80°C.

### 2.2 Determination of MICs

The broth micro-dilution method was used to determine the MICs of 8 antimicrobial agents in accordance with CLSI guidelines (CLSI, 2020a). The antibiotics selected correspond to the molecules used in fish farming for the treatment of furunculosis. All tests were performed in 96-well microplates. The plates were formatted in 8 identical series of 10 two-fold dilutions for florfenicol (32 to 0.06 mg/L), oxolinic acid (32 to 0.06 mg/L), flumequine (32 to 0.06 mg/L), enrofloxacin (2 to 0. 004 mg/L), oxytetracycline (64 to 0.125 mg/L), doxycycline (32 to 0.06 mg/L), sulfadiazine (256 to 0.5 mg/L) and the combination of sulfadiazine + trimethoprim (154/8 to 0.3/0.016 mg/L). The antibiotics (Sigma-Aldrich France, Saint Quentin Fallavier) were certified for purity and dissolved in solvents specific to each molecule (concentration of stock solutions equal to 5120 mg/L for all antibiotics except the combination sulfadiazine + trimethoprim concentrated at 6160 mg/L).

On removal from storage at −80°C, ASS strains were streaked onto TSA agar and incubated at 22°C ± 1°C for 48–72 h. Colonies of ASS were then inoculated onto Mueller Hinton agar and incubated at 22°C ± 1°C for 12 h. From this stationary phase broth, a new culture was initiated approximately 3 h before inoculation of the 96-well microplates, in order to obtain an exponential growth phase. The suspension was adjusted to approximately 10^6^ CFU/mL, using both the optical density and appropriate dilutions. Fifty microliters of the suspension were added to each well of the microplate. Four wells were used as positive controls (bacterial suspension only), and eight as negative controls (uninoculated MH broth medium only, using the medium used for antibiotic dilutions and the medium used for strain dilution for plating). The microplates were incubated at 22°C ± 1°C for 48 h. *A. salmonicida* subsp *salmonicida* ATCC 33658 was used as a quality control. For all ASS isolates tested, the inoculum density was verified by inoculation of MH agar through a Steers replicator (Sigma-Aldrich France, Saint Quentin Fallavier) with 1 μL of the suspension from the positive control well before incubation of the microplate.

### 2.3 MIC analysis

MIC_50_ and MIC_90_ were calculated from the distribution of MICs obtained. MIC_50_ is the MIC value at which ≥50% of the isolates in a test population are inhibited. MIC_90_ is the MIC value at which ≥90% of the strains within a test population are inhibited (Schwarz et al., 2010). The abbreviations ECV and ECOFF refer to consensus-based epidemiological cut-off values from CLSI and EUCAST, respectively. To date, no ECOFF has been defined by EUCAST for this bacterium. Oxolinic acid, florfenicol, oxytetracycline and the combination of sulfadimethoxine + ormetoprim are the only antibiotics for which ECVs have been determined by CLSI in *A. salmonicida* (including diverse subpopulations, *i.e.*, typical as well as some atypical strains), based on data from different laboratories and different countries (Miller and Reimschuessel, 2006; Smith et al., 2024). Our data were analyzed in relation to the ECVs reported by CLSI for these antibiotics. In table 1, white fields represent the tested dilution range, while gray fields represent untested concentrations. Strains with MIC values below the tested concentration range were assigned to the prior gray two-fold dilution box. Strains with MIC values above the tested concentration range were assigned to the next gray two-fold dilution box.

**TABLE 1:** Distribution of MICs (mg/L) in 406 isolates of ASS and interpretative criteria.

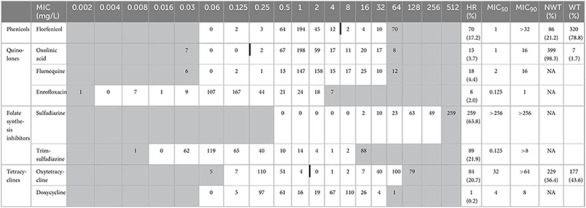

White fields represent the range of dilutions tested, whereas gray fields represent non-tested concentrations. Strains with MIC values below the tested concentration range were assigned to the prior two-fold dilution category. Strains with MIC values above the tested concentration range were assigned to the next two-fold dilution category. Thick vertical lines indicate epidemiological cut-off value (ECV) from CLSI when available. HR (%): number (and percentage) of isolates for which the MIC values were outside the range of concentrations tested, i.e., value below or above the range. NWT: number (and percentage) of non-wild type isolates i.e., with an MIC above the epidemiological cut-off value. WT: number (and percentage) of wild type isolates i.e., with an MIC below the epidemiological cut-off value. NA: Not Applicable, in the absence of an official epidemiological cut-off value. For the combination sulfadiazine + trimethoprim (noted Trim-sulfadiazine), the concentrations indicated are those of trimethoprim. The associated concentrations of sulfadiazine are approximately 19 times higher, in accordance with the CLSI procedure. For example, for a concentration of 0.125 mg/L of trimethoprim, the concentration of sulfadiazine was 2.4 mg/L.

### 2.4 Provisional epidemiological cut-off values determination and analysis

For the sake of clarity, the abbreviation CO_*Epid*_ is used throughout the study to designate the provisional epidemiological cut-off values obtained as part of this study. Our provisional CO_*Epid*_ were statistically determined according to two methods. The first method is designated the “NRI method” (Normalized Resistance Interpretation) as proposed by Kronvall (2010). The epidemiological cut-off value was obtained through the automatic excel programme available by courtesy of P. Smith, W. Finnegan and G. Kronvall, used with permission from the patent holder, Bioscand AB, TÄBY, Sweden (European patent No 1383913, US Patent No. 7,465,559). The second method is designated “ECOFFinder method” as proposed by Turnidge et al. (2006). The estimation was obtained using nonlinear regression on expanding subsets, through the 2020 updated version of the automatic excel programme available by courtesy of J. Turnidge, used with permission from EUCAST. For this method, the provisional ECOFF defined with lower percentages provide better sensitivity for non-wild type selection, while higher percentages provide better specificity for the wild-type population (see Table 2).

**TABLE 2:** Co_Epid_ (mg/L) values for ASS obtained by “NRI method” or by “ECOFFinder method.”

	NRI method		Provisional ECOFF values obtained using ECOFFinder					ECV (CLSI)	BPC (CLSI)	CO_Epid_
	**CO_WT_**	**sd**	**95%**	**97.5%**	**99%**	**99.5%**	**99.9%**			
**Phenicols**										
Florfenicol	4	0.79	2	2	2	2	4	**4**		**4**
**Quinolones**										
Oxolinic acid	4	0.83	2	2	2	4	4	**0.12**	**0.12**	**4**
Flumequine	4	0.79	4	4	4	4	4			**4**
Enrofloxacin	0.5	0.85	0.25	0.25	0.25	0.5	0.5			**0.5**
**Antifolates**										
Trim-sulfadiazine	0.25/4.8	0.84	0.25/4.8	0.25/4.8	0.25/4.8	0.5/9.6	0.5/9.6			**0.25/4.8**
**Tetracyclines**										
Oxytetracycline	1	0.68	0.5	0.5	0.5	1	1	**1**	**1**	**1**
Doxycycline	1	0.64	0.5	0.5	0.5	1	1			**1**

CO_WT_, cut-off wild-type obtained through the “NRI method”; Sd, standard deviation; ECOFF, epidemiological cut-off value obtained through the “ECOFFinder method” as defined by EUCAST; ECV, official epidemiological cut-off values set by CLSI. Only some ECVs have been defined by CLSI for ASS. BPC, official clinical breakpoint set by CLSI and used to provide a classification of projected clinical outcome of animal treatment based on the MIC, taking into account the expected exposure of the bacteria to the drug at a standard dose. Only 2 BPC are currently available for ASS. CO_Epid_, provisional epidemiological cut-off value proposed by this study.

The susceptibility of the strains to different families of antibiotics, *i.e*., with different modes of action, was also assessed. To compare the susceptibility of each strain to different families of antibiotics, we proceeded as follows: a strain was considered to belong to the NWT category to a particular family of antibiotics if it fulfilled the following conditions: (1) for phenicols, a MIC value above the ECV for florfenicol (4 mg/L); (2) for tetracyclines, a MIC value above both the ECV for oxytetracycline (1 mg/L) and the CO_*Epid*_ for doxycycline (1 mg/L); (3) for quinolones, a MIC value above the corresponding CO_*Epid*_ for oxolinic acid (4 mg/L) [be careful, not the ECV defined by CLSI] and flumequine (4 mg/L), and enrofloxacin (0.5 mg/L); (4) for the combination trimethoprim + sulfadiazine, a MIC value above the CO_*Epid*_ (0.25/4.8 mg/L).

### 2.5 Statistical analysis

The strains were collected over several years, and originate from different regions of France. The evolution of the proportion of strains with MIC above CO_*Epid*_ was observed as a function of year of sampling and region of origin by statistical regression. The Odds Ratios and their Confidence Intervals were calculated using R Studio software version 2023.12.1.402 (R Studio: integrated development for R, PBC, Boston) for R (R Core Team, 2021, R Foundation for Statistical Computing, Vienna, Austria).

## 3 Results

For each MIC determination assay, the reference strain and inoculum density data were in accordance with CLSI recommendations (data not shown). 415 strains were collected and tested, of which 406 had complete MIC values. When the MIC determination was considered unsatisfactory because the bacterial enumeration was outside the CLSI standards or because the MIC values were abnormal for at least one antibiotic, a new complete MIC determination was performed a maximum of 3 times. A total of 9 strains were finally discarded from the collection.

The MIC distribution of 406 ASS isolates for eight antimicrobial agents is shown in Table 1, together with the corresponding MIC_50_ and MIC_90_. MIC values above the tested range were observed for all antimicrobials (HR), in particular for sulfadiazine (around 64% of the strains), oxytetracline (around 20% of the strains) and florfenicol (around 17% of the strains). These strains displaying very high MIC can be considered as highly resistant to the tested antibiotics. Furthermore, except for doxycycline, the difference between MIC_50_ and MIC_90_ was at least three dilutions, and often much higher, illustrating the high proportion of acquired resistance in ASS strains of fish origin in France.

The proportion of wild-type (WT) strains in the ASS population tested can be determined using the ECVs available for three antibiotics (oxolinic acid, oxytetracycline and florfenicol). Almost none of the ASS responsible for furunculosis from our collection belongs to the wild-type population for oxolinic acid, only about 44% for oxytetracyclines, but about 79% for florfenicol. This reflect a wide variation in the epidemiological situation.

CO_*Epid*_ were calculated from our data for seven antimicrobials, whether or not an ECV was available, and are presented in Table 2. No value was computed for sulfadiazine due to the high MIC values and the proportion of strains with MICs outside the concentration range tested. For all antimicrobials tested, the MIC standard deviation never exceeded 1.2 log2 mg/L, the limit implemented in the NRI method spreadsheet (Kronvall, 2010). Similar CO_*Epid*_ values were obtained by the two methods for all antibiotics tested, with differences of only one dilution step. Our provisional CO_*Epid*_ values correspond to those obtained by the NRI method. The provisional epidemiological cut-off values calculated from our data were identical to the ECV defined by CLSI for florfenicol (4 mg/L) and oxytetracycline (1 mg/L). However, the CO_*Epid*_ calculated for oxolinic acid was very different, ranging from 0.12 mg/L (ECV) to 4 mg/L (CO_*Epid*_).

For the quinolones, our CO_*Epid*_ of 4 mg/L is identical for oxolinic acid and flumequine. The CO_*Epid*_ for enrofloxacin is lower: 0.5 mg/L. Based on our CO_*Epid*_, approximatively 14, 16, and 12% of the tested ASS would be classified as NWT for oxolinic acid, flumequine and enrofloxacin, respectively. A comparison of the susceptibility to quinolones of each strain revealed that 95% of strains with an MIC above the CO_*Epid*_ for oxolinic acid also exhibited a MIC above the CO_*Epid*_ for flumequine. Likewise, 90% of strains with a MIC above the CO_*Epid*_ (0.5 mg/L) for enrofloxacin had a MIC above the corresponding CO_*Epid*_ for flumequine (4 mg/L) and oxolinic acid (4 mg/L). The MIC values for ASS were very consistent between these different quinolones, reflecting a cross-resistance mechanism.

For oxytetracycline, a total of 98 % of the strains identified as non-wild type (*i.e.*, with an MIC > ECV = CO_*Epid*_ = 1 mg/L) exhibited MIC values above our CO_*Epid*_ for doxycycline (CO_*Epid*_ = 1 mg/L), reflecting a cross-resistance mechanism. A comparison of the MIC values of NWT strains (*i.e.*, with MIC values above the CO_*Epid*_ = 1 mg/L) between the two tetracyclines revealed that the MIC values obtained for doxycycline were systematically lower than those obtained for oxytetracycline. Moreover, in 89% of cases, the discrepancy in MIC values between the two tetracyclines was at least three dilutions. Nevertheless, this outcome was not observed in strains with MICs below the CO_*Epid*_.

A total of 242 ASS strains belonged to the NWT category of at least one antibiotic family (as defined in the Materials and Methods section), about 60% of our 406 strains. Of these, 21 strains (5%) appeared to have reduced susceptibility (i.e., MIC > CO_*Epid*_) to 2 antibiotic families, 87 (21%) to 3 families and 12 strains (3%) were classified as non-wild type for all antibiotics tested.

**FIGURE 1:**
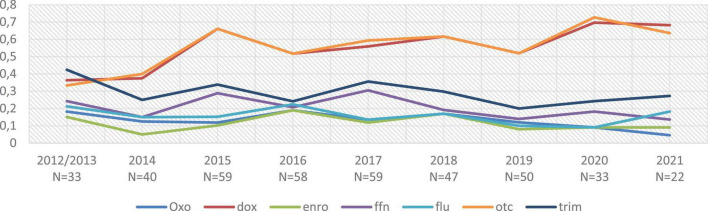
Evolution of the proportion of NWT strains over time. NWT (non-wild type) strains are defined as strains with an MIC above our CO_Epid_ threshold; oxo, oxolinic acid; dox, doxycycline; enro, enrofloxacin; ffn, florfenicol; flum, flumequine; otc, oxytetracycline; trim, sulfadiazine + trimethoprime combination. N, number of strains isolated on that year. A total of 5 strains where sampling date was not formally confirmed were removed from the dataset.

Although this study is not a true prevalence study, but rather an event-based epidemiological study, the evolution of the proportion of NWT strains (*i.e.*, strains with MIC > CO_*Epid*_) was observed as a function of year of sampling and region of origin by statistical regression (data not shown). Thus, the proportion of NWT strains within the analyzed strains decreased when the sample originated from Brittany, for oxolinic acid (OR = 0.53, confidence interval (CI) of the odds ratio (OR) equal to [0. 3–0.93]), for oxytetracycline (OR = 0.21, CI-OR = [0.13–0.34]), and for doxycycline (OR = 0.21, CI-OR = [0.13–0.33]), whereas it increased for florfenicol (OR = 1.68, CI-OR = [1.01–2.79]). The proportion of NWT strains was stable over time in our dataset, except for the tetracycline family, where this proportion has increased in the last years, from about 50% NWT strains in the early years [2012–2016] to about 60% in the final years [2018–2021] (see Figure 1). All raw results, including year of sampling and geographical origin of the strains, are available in Supplementary Data.

## 4 Discussion

### 4.1 Relevance of the ASS collection

A total of 406 isolates of ASS were collected from two diagnostic laboratories specializing in bacterial diseases of fish. These laboratories are located in two of the main trout producing regions of France, Brittany and New Aquitaine. The geographical origin of our strains is therefore not homogeneous across France, reflecting the location of our partner laboratories. Around 58% of our strains originated from the Brittany region, which accounts for 20% of French trout production, and 24% from the New Aquitaine region, which accounts for 30% of French trout production (Agreste-French Minitry of Agriculture, 2024). Our selection of strains was highly targeted to ASS and aimed to limit the possibility of misidentifications. Firstly, all strains had been obtained from samples collected by expert veterinarians from fish showing clinical signs of furunculosis. *Aeromonas salmonicida* is one of the oldest known pathogens of fish, particularly salmonids (Roberts, 2012). Historically, their classification has been based on the source of isolation: from salmonids (such as trout) they are classified as “typical,” from non-salmonid hosts or environmental samples they are classified as “atypical” strains (Vasquez et al., 2022). As all our strains were obtained from diseased farmed trout, it is reasonable to assume that the vast majority, if not all, of our strains are typical *A. salmonicida* (also known as ASS) (Roberts, 2012). However, only sequencing of each strain could guarantee this. Within our partner laboratories, strains that do not originate from diseased fish (strains of food origin, environmental origin, other animal species, or even unspecified) have an identification that is limited to the genus level (*Aeromonas sp*). Our study did not collect these strains. Secondly, the bacteria were identified as ASS by laboratories specializing in aquaculture bacteriology. In current practice in these veterinary laboratories, identification to the species level is achieved by a combination of bacterial culture characteristics, MALDI-TOF scoring, and clinical signs reported by veterinarians. The following species or subspecies are at risk of misidentification: *Aeromonas salmonicida pectinolytica*, *Aeromonas salmonicida masoucida*, *Aeromonas eucrenophila*, *Aeromonas bestiarum*, and, to a lesser extent, *Aeromonas popoffi*, *Aeromonas encheleia*. As MALDI-TOF alone has recently been reported to be insufficient for accurate species-level identification of *Aeromonas* isolates (Pérez-Sancho et al., 2018; Kitagawa et al., 2022), this combination reduces the risk of misidentifying strains, at least until sequencing becomes routinely available for diagnostic purposes. Thirdly, each isolate included in this study, obtained from the ASS collections of the partner laboratories, was streaked on receipt to ensure isolate purity, and its identity was confirmed by culture characteristics and the PCR method of Byers et al. (2002). None of the strains received showed characteristics that would question their species identification. We are therefore very confident in the quality of the identification of ASS in this study.

Our 406 strains come from 96 different farm companies, with strains collected over a 10-years period. Thus, some of the strains in our set originate from the same farm company and may be epidemiologically related. However, a given company may operate several ponds, and several separate operations (hatcheries, nurseries, grow-out), sometimes in very distant locations, making it difficult to assess the epidemiological link, as all strains are registered by the veterinary laboratory under the same company name. Although most farm companies submitted samples to the partner laboratory less than five times during the study period (2012–2021), a few farms provided between 20 and 40 samples each. The specific antibiotic susceptibility pattern of ASS on these farms could skew the observed global distribution of MICs in ASS strains, particularly if a high prevalence of furunculosis has led to extensive antibiotic use on these farms. However, a comparative study of strains from the same company showed no obvious association. The large number of strains tested in our study may limit the potential bias of testing isolates that may be epidemiologically related. Furthermore, this potential bias should not affect the determination of CO_*Epid*_. Detailed information on the origin of each strain can be found in the Supplementary Data.

To our knowledge, this is the largest antibiotic susceptibility study of ASS ever conducted. However, the proportion of NWT stains in this study does not necessarily reflect the actual prevalence encountered in commercial fish farms, as there was no random sampling. Strains were collected by the laboratories on an event-driven basis, by isolating and identifying bacteria from samples taken from fish by veterinarians. It could be argued that the proportion of NWT strains in our database may be overestimated, because veterinarians are more likely to send bacteriological samples to the laboratories when treatment fails, which may lead to an over-representation of resistant strains in particular. However, in France, when furunculosis is observed in a trout farm, an antibiotic susceptibility test is requested in more than 80% of cases before antibiotics are used as first-line treatment, and in 100% of cases when antibiotics are used as second-line treatment (Carabin, 2022). Without claiming to reflect the true prevalence of antibiotic susceptibility in ASS strains, the large number of strains collected in this study provides a credible approximation of the distribution of antibiotic susceptibility in the trout industry in Western France. Considering that the farms are generally flow-through systems, with water being collected and discharged directly into watercourses without any specific treatment, these data provide an original picture of the epidemiological situation of antibiotic susceptibility in ASS, both from the point of view of improving aquaculture production practices (particularly with regard to the use of antibiotics) and in support of public health policies.

### 4.2 Quinolones

The MIC distribution appeared to be bimodal for quinolones, with two distinct populations identified for oxolinic acid (1st generation quinolone), flumequine (2nd generation quinolone) or enrofloxacin (3rd generation quinolone). Enrofloxacin has been classified as a critical molecule under French regulation since 2016. As a result, its use in fish farming has been very limited, unlike the other quinolones. Oxolinic acid and flumequine have been used to treat furunculosis in salmonids in France since the 1980s. These molecules, together with oxytetracycline, are still the only ones with a marketing authorization (MA) for use in fish in France. This means that they have to be used preferentially. However, to date in France, the antibiotics used as first-line treatment for furonculosis in trout farms are mainly florfenicol (38% of veterinary drugs prescribed) and the combination of sulfadiazine + trimethoprim (31% of veterinary drugs prescribed), drugs without a MA for use in fish (Carabin, 2022). The NWT strains identified by our CO_*epid*_ for a given quinolone were practically all classified as NWT for the other quinolones. This finding is consistent with current knowledge on quinolones, particularly fluoroquinolones, where resistance to one agent often leads to resistance to all agents of this class (Goñi-Urriza et al., 2000; Naviner et al., 2011; Giguère and Dowling, 2013). Thus, the use of first- or second-generation quinolones in fish farming may contribute to the selection of ASS resistant to the latest generation fluoroquinolones. Indeed, the European Medicines Agency has placed this entire family into “category B – restricted” (EMA, 2019). This category is intended for antibiotics that are of critical importance in human medicine and whose use in animals should be restricted in order to reduce the risk to public health by giving preference, when possible, to antibiotics in “category C – caution” or in “category D – prudence”, and by testing for antimicrobial susceptibility whenever possible. To date, only enrofloxacin has been classified under the French veterinary legislation as being subject to specific restrictions on prescription by veterinarians.

The CLSI established an ECV for oxolinic acid in *Aeromonas salmonicida* of 0.125 mg/L in VET04Ed3 (CLSI, 2020b), which is much lower than the value measured in our study (4 mg/L). In 2024, by aggregating the data used by CLSI to define the ECV for oxolinic acid (Miller and Reimschuessel, 2006) with new data from various laboratories, a new provisional epidemiological cut-off lowered to 0.0625 was proposed (Smith et al., 2024). The MIC_50_ can be used as an estimate of the susceptibility of a particular population of bacteria to a particular antibiotic. Thus, it is important to note that the MIC_50_ of oxolinic acid calculated in our study (1 mg/L) was close to that previously reported (2 mg/L) on 74 isolates collected from infected fish in France during the period 1985–2020 (Baron et al., 2021). However, it was much higher than that reported in other studies on other fish species, or in water samples from the environment (Tsoumas et al., 1989; Barnes et al., 1990, 1991; Miller and Reimschuessel, 2006; Baron et al., 2017; Scarano et al., 2018) – see Table 3. The disappearance of the WT population of ASS from French trout farms, as already suggested by the MIC_50_ data presented, is the most likely explanation for this discrepancy between the international epidemiological cut-off values (ECV) and the provisional cut-off calculated in this study (CO_*Epid*_), with a rightward shift of MIC values in the French ASS subpopulation associated with farmed trout.

**TABLE 3:** Comparison of the MIC_50_, MIC_90_ and CO_WT_ values reported in the literature for the 3 generations of quinolones tested in our study.

Drug	MIC_50_	MIC_90_	CO_WT_	Strains collected	References
**Oxolinic acid**	**1**	**16**	**4**	**406 ASS from diseased trout in France collected between 2012 and 2021**	**This study**
	2	>8	ND	73 *A. salmonicida* from diseased trout in France during period 1985–2020	Baron et al., 2021
	0.03	1	0.125	217 typical and atypical *A. salmonicida* isolates from 12 countries (the USA accounted for half of the isolates) and from 28 fish species between 1955 and 2004	Miller and Reimschuessel, 2006
	0.016	0.016	0.031	45 *A. salmonicida* from environmental waters in France collected in 2014	Baron et al., 2017
	0.06	16	0.25	104 strains of *Aeromonas* spp. (including several species) sampled from sea bream reared in Italian mariculture farms	Scarano et al., 2018
	0.03	0.4	ND	45 oxolinic acid susceptible *A. salmonicida* collected mainly from natural outbreaks of furunculosis on salmonid frams in Scotland in the 1990s	Barnes et al., 1990
	0.007	2	ND	70 *A. salmonicida* isolates from diseased fish (different species) mainly from UK	Tsoumas et al., 1989
**Flumequine**	**2**	**16**	**4**	**406 ASS from diseased trout in France collected between 2012 and 2021**	**This study**
	0.031	0.062	0.062	45 *A. salmonicida* from river water in France sampled in 2014	Baron et al., 2017
	0.12	16	0.25	104 strains of *Aeromonas* spp. (including several species) sampled from sea bream reared in Italian mariculture farms	Scarano et al., 2018
	0.075	0.1	ND	45 oxolinic acid susceptible *A. salmonicida* collected mainly from natural outbreaks of furunculosis on salmonid frams in Scotland in the 1990s	Barnes et al., 1991
**Enrofloxacin**	**0.125**	**1**	**0.5**	**406 ASS from diseased trout in France collected between 2012 and 2021**	**This study**
	0.25	1	ND	151 *A. salmonicida* from diseased fish in France	Viel et al., 2021
	0.25	8	0.06 / 0.125	556 *Aeromonas* spp. (mainly *A. veronii*) from fish, water or sediments in China	Lin et al., 2022
	0.016	0.031	0.031/0.062	45 *A. salmonicida* from river water in France sampled in 2014	Baron et al., 2017
	0.02	0.05	ND	45 oxolinic acid susceptible *A. salmonicida* collected mainly from natural outbreaks of furunculosis on salmonid frams in Scotland in the 1990s	Barnes et al., 1990
**Ciprofloxacin**	0.0156	0.0156	ND	217 strains of *Aeromonas* spp. (only 5% *A. salmonicida*) of various origins	Kämpfer et al., 1999
	0.2	0.5	ND	81 nalidixic acid-resistant strains of *Aeromonas* spp. from river water in Spain or France sampled in 1996	Goñi-Urriza et al., 2000
	<0.5	<0.5	ND	79 motile *Aeromonas* spp. (mainly *A. hydrophila*) collected from water samples from 10 fish farms (African catfish and Dutch eel) in the Netherlands	Penders and Stobberingh, 2008
	0.008	0.25	ND	234 *Aeromonas* spp. (mainly *A. hydrophila*) from 3 medical centers (mainly from human blood) in Taiwan	Ko et al., 1996

MIC_50_, MIC value at which growth is inhibited in ≥50% of the isolates in a test population; MIC90, MIC value at which growth is inhibited in ≥90% of the isolates within a test population; CO_WT_, provisional epidemiological cut-off value calculated within the study population; ND, not determined in the study.

There is limited literature on ASS susceptibility to flumequine, and no EUCAST or CLSI defined epidemiological cut-off value. However, as for oxolinic acid, our calculated MIC_50_ for flumequine (2 mg/L) is much higher than those reported in the literature (Barnes et al., 1991; Baron et al., 2017; Scarano et al., 2018) – see Table 3. Some authors have proposed provisional epidemiological cut-off values ranging from 0.06 mg/L to 0.25 mg/L, far from our CO_Epid_ of 4 mg/L (Baron et al., 2017; Scarano et al., 2018). Comparison of our data with the literature and evidence of cross-resistance within this antibiotic family suggests that the WT population of ASS to flumequine has also disappeared from trout farms in western France. In light of our results, there is an urgent need to re-evaluate the current recommended dosing regimen for the use of first- and second-generation quinolones, which was established more than 30 years ago, as the dosing regimen for an antibiotic is based on the bacterial population that is sensitive to that antibiotic. Similarly, the clinical breakpoints used for antibiotic susceptibility testing should be re-evaluated, at least in France.

There is no EUCAST or CLSI defined epidemiological cut-off value for enrofloxacin. The MIC_50_ values found in the literature for enrofloxacin, or its metabolite ciprofloxacin, are split between values around our own MIC_50_ of 0.125 mg/L (Goñi-Urriza et al., 2000; Penders and Stobberingh, 2008; Baron et al., 2017; Viel et al., 2021; Lin et al., 2022), and values that are 10 times lower (Barnes et al., 1990; Ko et al., 1996; Kämpfer et al., 1999; Baron et al., 2017) – see Table 3. However, it is still difficult to identify ASS at the species level, and many of those studies report MIC_50_ in sets of isolates of *Aeromonas* identified at the genus level only. This is an important limitation in the attempt to set epidemiological cut-off values in clinically important *Aeromonas* species. Nevertheless, although we are very confident in our CO_epid_ thanks to our high confidence in species level identification, it remains possible that the WT population has disappeared from trout farms in France, as is probably the case with first and second generation quinolones. Only 4.4% of the strains collected in our study showed an MIC of 0.03 mg/L or less, the provisional epidemiological cut-off value for *Aeromonas salmonicida* from river water suggested by Baron et al. (2017). Enrofloxacin displays a concentration-dependent bactericidal activity against Gram-negative bacteria. To optimize the efficacy of this antibiotic while limiting the selection of resistant bacteria, PK/PD (pharmacokinetic/pharmacodynamics) modeling was carried out on the dosing regimen for rainbow trout (Viel et al., 2021). When this model is compared with our sensitivity data for ASS, the preferred dosing regimen would be between 40 and 80 mg/kg, which is completely unrealistic in the aquaculture sector in terms of cost, production of the medicated feed, palatability (the standard dose is 10 mg/kg) or residues in tissues intended for human consumption. Therefore, the use of enrofloxacin to treat ASS in France may no longer be appropriate, even after antibiotic susceptibility testing, while under-dosing may lead to selection of resistant bacteria.

### 4.3 Other antibiotics

#### 4.3.1 Phenicol

Florfenicol is the most widely used antibiotic in fish farming in France (Carabin, 2022). Our CO_Epid_ corresponds to the ECV defined by CLSI (4 mg/L) (CLSI, 2020b) and is similar to other provisional epidemiological cut-off values described in the literature, with a difference of maximum one drug dilution (Miller and Reimschuessel, 2006; Baron et al., 2017; Scarano et al., 2018; Duman et al., 2020; Baron et al., 2021; Smith et al., 2024). Almost 20% of the strains in our collection are highly resistant to this antibiotic (MIC > 16 mg/L). Therefore, the systematic use of antimicrobial susceptibility testing in fish farming is recommended.

#### 4.3.2 Sulfonamides and combinations with diaminopyrimidines

Around 76% of the strains collected from trout farms in our study showed an MIC ≥ 256 mg/L, and can be considered as highly resistant to sulfadiazine alone. The same results were observed in Turkey, where 93 strains out of 98 *Aeromonas* spp. strains sampled from different fish species and locations had an MIC ≥ 256 mg/L (Duman et al., 2020), or in Spain and southern France, where 72% of the 138 *Aeromonas* spp. strains from river water had an MIC ≥ 256 mg/L (Goñi-Urriza et al., 2000). For now, it can be assumed that it is no longer possible to suggest treating ASS in fish with a sulfonamide alone. The combination of a diaminopyrimidine, such as trimethoprim, with a sulfonamide, in a 1:19 ratio, allows a synergistic interaction against bacteria, resulting in a lower MIC and a bactericidal effect. This synergistic effect has been confirmed *in vitro* for ASS, with very low MIC values compared to sulfadimidine alone. For the combination sulfadiazine + trimethoprim (SDT) in our collection, MIC distribution appeared to be trimodal, with a NWT population (MIC ≤ 0.25/4.8 mg/L – around 71% of the population), a moderately resistant population (around 7 % of the population) and a highly resistant population (MIC ≥ 4/77 mg/L – around 22% of the population). Our CO_Epid_ (0.25/4.8 mg/L) corresponds to other provisional epidemiological cut-off values described in the literature, for sulfamethoxazole + trimethoprim (Baron et al., 2017; Lin et al., 2022) and is close to the ECV defined for sulfadimethoxine + ormetoprim (0.5/9.5 mg/L) (Miller and Reimschuessel, 2006; CLSI, 2020b; Smith et al., 2024).

The good safety and the very low cost of this combination explain the importance of this antibiotic family in fish farming today, provided an antibiogram is carried out for ASS treatment. In recent years, however, the relevance of this combination *in vivo* has been questioned. Antibacterial diaminopyrimidines are combined with a variety of sulfonamides in a fixed ratio (1:5) in the marketed drugs, which in humans results in a 1:19 ratio of drug concentrations in plasma after oral or parenteral administration (Prescott, 2013). Veterinary preparations are combined in the same fixed 1:5 ratio. However, this ratio was a direct transposition of what was known in human medicine, and was not established by taking into account the pharmacokinetics of these drugs in animals. Considering the low susceptibility of *Aeromonas* to sulfadiazine alone, and to trimethoprim alone (Kämpfer et al., 1999; Penders and Stobberingh, 2008; Scarano et al., 2018), it seems appropriate to re-evaluate the current dosage regimen currently used in fish, even though the half-lives of sulfadiazine and trimethoprim appear to be fairly long in trout (Salte and Liestøl, 1983; Tan and Wall, 1995).

#### 4.3.3 Tetracyclines

Oxytetracycline (OTC), a first-generation (natural) tetracycline, and doxycycline (DOX), a second-generation (semi-synthetic) tetracycline, have a bimodal MIC distribution. Our CO_Epid_ (1 mg/L), which is the same for both molecules, corresponds to the ECV defined by CLSI for OTC (CLSI, 2020b), and is similar to other provisional epidemiological cut-off values described in the literature, for OTC (Scarano et al., 2018; Smith et al., 2024) and for DOX (Lin et al., 2022). As expected, the antibacterial potency of doxycycline is greater, resulting in a MIC value systematically lower than that obtained for OTC by at least 3 dilutions, but mainly in the NWT population. This difference was not found in the WT population, which was more surprising and results in the same CO_Epid_ for both antibiotics. Doxycycline could have been a relevant alternative to OTC, which has very low bioavailability in trout (Viel et al., 2024) and a very high prevalence of resistance in ASS. However, the high cost of this drug compared to OTC, and an epidemiological cut-off similar to OTC, make this alternative less interesting in fish farming.

### 4.4 Perspectives

Few consensus-based epidemiological cut-off values for *Aeromonas salmonicida* have been defined by CLSI. The European Committee on Antimicrobial Susceptibility Testing website^1^ provides MIC distributions for *A. caviae*, *A. hydrophila* and *A. veronii*, but no ECOFFs have been proposed due to the small number of observations, and no data are available for *A. salmonicida*. This work is therefore a contribution to the understanding of this bacterium.

Furunculosis is a relatively common disease in trout farms, and its prevalence is likely to increase with global warming. To control the disease, antibiotics are sometimes required in flow-through aquaculture farms, at the crossroads of animal, human and environmental health. The availability of such data on the epidemiology of ASS in France contributes to the monitoring of antibiotic resistance in a bacterial genus often considered to be a good indicator of antibiotic susceptibility in the aquatic environment (Goñi-Urriza et al., 2000; Alcaide et al., 2010; Lamy et al., 2022). In particular, *Aeromonas* spp. are especially able to promote interactions and gene exchange between One Health components (Lamy et al., 2022). These data are also essential for defining appropriate dosing regimens for antibiotics in fish. Indeed, in aquaculture veterinary medicine, a number of antibiotics are used off-label, with dosages defined several decades ago. A good definition of the susceptibility of ASS strains is a key element in reassessing dosage regimens for treatment. Finally, these data are essential for determining clinical breakpoints, which are crucial for interpreting antibiograms, to help prescribers make better use of veterinary medicines.

## Data Availability

The original contributions presented in this study are included in this article/Supplementary material, further inquiries can be directed to the corresponding author.

## Footnotes

1
www.eucast.org
