# Supplementary material for: Susceptibility of Aeromonas salmonicida subsp. salmonicida bacteria from French farmed trout to antibiotics commonly used in fish farming, and attempt to set epidemiological cut-off values
Source: Front Microbiol. 2025 Mar 11;16:1532748. doi: 10.3389/fmicb.2025.1532748 (PMC11932985; doi:10.3389/fmicb.2025.1532748)
Supplement: Supplementary file 1 [file Data_Sheet_1.pdf]

Fish farm company	Region of sampling	Sampling date	FLOR	OXO	FLU	ENRO	SZ	SZ-TRI		OTC	DOX
F01	Brittany	Sep-2015	**>32**	1	1	0.125	128	0.03	0.6	0.25	0.25
F02	Brittany	Jun-2018	1	1	2	0.125	**>256**	0.06	1.2	0.5	0.25
F03	Brittany	Aug-2021	1	1	2	0.125	**256**	0.03	0.6	0.25	0.5
F04	Other area	Mar-2013	1	2	2	0.125	**>256**	0.125	2.4	0.5	0.5
	Other area	Mar-2013	1	1	2	0.25	**>256**	0.125	2.4	0.5	0.5
	Other area	Mar-2013	4	2	4	0.25	**>256**	0.5	9.625	0.5	0.5
F05	Other area	Jan-2019	0.5	0.5	<0.06	0.008	**256**	0.03	0.6	0.25	0.25
F06	Brittany	Jul-2012	1	1	2	0.125	**256**	0.06	1.2	0.5	0.25
	Brittany	Aug-2014	0.5	1	2	0.06	128	0.06	1.2	0.5	0.25
	Brittany	Aug-2014	**>32**	0.5	1	0.06	**>256**	**>8**	**>154**	**>64**	4
	Brittany	Aug-2014	2	4	8	0.5	128	0.06	1.2	0.5	1
	Brittany	Oct-2014	**>32**	1	1	0.125	**>256**	**>8**	**>154**	32	8
	Brittany	Sep-2015	**>32**	2	4	0.25	**>256**	**>8**	**>154**	**>64**	8
	Brittany	Sep-2015	**>32**	1	2	0.125	**>256**	**>8**	**>154**	**64**	8
	Brittany	Oct-2015	**>32**	1	1	0.125	**>256**	**>8**	**>154**	**64**	4
	Brittany	Nov-2015	**>32**	1	1	0.125	**>256**	**>8**	**>154**	**64**	4
	Brittany	Nov-2015	**32**	1	8	0.5	**>256**	**>8**	**>154**	32	2
	Brittany	Aug-2016	**>32**	0.5	1	0.06	**>256**	**>8**	**>154**	32	4
	Brittany	Aug-2016	**>32**	1	2	0.06	**>256**	**>8**	**>154**	**64**	4
	Brittany	Oct-2016	**>32**	16	16	**2**	**>256**	**>8**	**>154**	**>64**	16
	Brittany	Aug-2017	**32**	1	1	0.06	**>256**	**>8**	**>154**	32	4
	Brittany	Aug-2017	**>32**	0.5	1	0.06	**>256**	**>8**	**>154**	**64**	4
	Brittany	Aug-2017	16	0.5	1	0.06	**>256**	**>8**	**>154**	16	1
	Brittany	Aug-2017	1	1	2	0.125	64	0.06	1.2	0.5	0.5
	Brittany	Sep-2017	**>32**	1	2	0.125	**>256**	**>8**	**>154**	**64**	8
	Brittany	Jul-2018	**>32**	1	1	0.06	**>256**	**>8**	**>154**	**64**	2
	Brittany	Sep-2019	1	1	2	0.125	128	0.03	0.6	0.25	0.5
	Brittany	Sep-2019	**>32**	2	2	0.125	**>256**	**>8**	**>154**	**64**	4
	Brittany	Aug-2020	**>32**	1	1	0.125	**>256**	**>8**	**>154**	32	4
	Brittany	Sep-2020	**>32**	1	2	0.125	**>256**	**>8**	**>154**	32	4
	Brittany	Aug-2021	**>32**	1	1	0.06	**>256**	**>8**	**>154**	**64**	8
	Brittany	Aug-2021	**32**	4	8	0.5	**>256**	**>8**	**>154**	**64**	4
F07	Brittany	Jun-2012	1	1	1	0.06	64	0.06	1.2	<0.125	0.25
	Brittany	Oct-2012	**>32**	8	2	0.5	**>256**	**>8**	**>154**	32	2
	Brittany	Nov-2013	1	2	4	0.25	**256**	0.06	1.2	0.25	0.5
	Brittany	May-2014	0.5	0.5	1	0.06	128	0.03	0.6	0.5	0.25
	Brittany	Jul-2014	0.5	2	2	0.25	**256**	0.03	0.6	0.25	0.25
	Brittany	Aug-2015	1	1	2	0.25	**256**	0.06	1.2	0.5	0.25
	Brittany	Jul-2016	1	1	2	0.25	128	0.03	0.6	0.5	0.5
	Brittany	Aug-2016	0.5	1	1	0.125	32	0.06	1.2	0.25	0.25
	Brittany	Jun-2018	1	1	2	0.125	128	0.06	1.2	0.5	0.25
F08	Brittany	Jun-2015	1	0.5	1	0.06	128	0.06	1.2	0.25	0.25
F09	Brittany	Nov-2013	2	4	8	0.5	128	0.06	1.2	0.5	1
F10	Other area	Jul-2016	2	4	8	1	32	0.06	1.2	1	2
F11	Brittany	Jun-2019	1	0.5	1	0.125	**256**	0.03	0.6	0.5	0.25
F12	Brittany	Oct-2013	1	2	2	0.25	**>256**	0.125	2.4	0.25	0.5
	Brittany	Oct-2013	1	1	1	0.125	128	0.06	1.2	0.25	0.5
	Brittany	Nov-2013	1	2	2	0.125	128	0.06	1.2	0.25	0.5
	Brittany	May-2014	0.5	0.5	1	0.06	128	0.03	0.6	0.125	0.25
	Brittany	Aug-2014	0.5	1	1	0.125	128	0.06	1.2	0.5	0.25
	Brittany	Oct-2014	0.25	2	1	0.06	**256**	0.06	1.2	<0.125	0.25
	Brittany	Jan-2015	0.5	1	1	0.06	128	0.06	1.2	<0.125	0.25
	Brittany	May-2015	2	1	1	0.125	**>256**	0.03	0.6	**>64**	8
	Brittany	Jul-2015	0.5	1	1	0.125	128	0.03	0.6	0.25	0.25
	Brittany	Sep-2015	0.5	1	1	0.125	128	0.06	1.2	0.25	0.25
	Brittany	Sep-2015	1	0.5	1	0.125	32	0.03	0.6	0.25	0.25
	Brittany	Sep-2016	1	0.5	1	0.06	64	0.06	1.2	0.5	0.5
	Brittany	Oct-2016	1	1	1	0.06	64	0.06	1.2	0.5	0.5
	Brittany	Jun-2017	0.125	1	1	0.03	32	0.03	0.6	0.125	0.125
	Brittany	Jun-2018	2	2	2	0.25	128	0.03	0.6	0.25	0.5
F13	Brittany	Jun-2019	0.5	1	2	0.125	**>256**	0.125	2.4	**>64**	4
F14	Brittany	Jul-2012	2	16	16	1	**>256**	0.25	4.8	**>64**	8
F15	Other area	NA	1	0.5	1	0.06	**>256**	0.25	4.8	**>64**	8
	Other area	NA	2	**>32**	16	1	**>256**	0.5	9.625	**>64**	4
	Other area	NA	0.5	0.5	0.5	0.06	**>256**	0.25	4.8	**64**	4
F16	New Aquitaine	Jul-2006	2	1	2	0.25	**256**	0.25	4.8	**64**	4
	New Aquitaine	Jul-2006	0.5	2	2	0.5	**256**	8	154	**64**	8

F17	Brittany	Sep-2015	**32**	1	1	0.125	**>256**	**>8**	**>154**	32	2
	Brittany	Sep-2017	**>32**	16	16	1	**>256**	**>8**	**>154**	**>64**	8
	Brittany	Jul-2018	1	1	2	0.125	**256**	0.06	1.2	0.25	0.25
F18	Brittany	Sep-2016	**>32**	16	8	1	128	2	38.5	16	8
	Brittany	Oct-2016	0.5	0.5	1	0.06	64	0.03	0.6	0.25	0.25
	Brittany	Jul-2017	1	0.5	1	0.06	128	0.03	0.6	0.25	0.25
F19	Brittany	Apr-2019	**>32**	2	2	0.125	**>256**	**>8**	**>154**	32	4
F20	Brittany	Dec-2019	2	**32**	**>32**	1	**>256**	**>8**	**>154**	**64**	16
F21	Other area	Jul-2017	1	0.25	0.125	<0.004	64	0.03	0.6	0.25	0.25
F22	Brittany	Dec-2020	4	4	4	0.25	**>256**	0.25	4.8	**64**	16
F23	New Aquitaine	Dec-2017	1	1	2	0.125	**>256**	0.25	4.8	0.25	0.25
	Other area	Jun-2018	1	2	2	0.125	128	0.06	1.2	0.25	0.25
F24	New Aquitaine	Jun-2015	1	0.5	2	0.125	**>256**	0.125	2.4	**64**	8
F25	Brittany	Aug-2015	0.5	1	1	0.125	128	0.06	1.2	0.25	0.25
	Brittany	Dec-2015	1	1	2	0.25	**>256**	0.06	1.2	0.25	0.25
	Brittany	Aug-2016	1	2	2	0.25	**>256**	0.125	2.4	0.5	0.5
	Brittany	Aug-2017	1	1	1	0.125	128	0.06	1.2	0.25	0.25
	Brittany	Aug-2018	1	1	2	0.125	**256**	0.06	1.2	0.25	0.25
	Brittany	Sep-2018	1	2	1	0.125	128	0.06	1.2	0.25	0.5
	Brittany	Jul-2019	1	1	2	0.06	128	0.06	1.2	0.5	0.5
	Brittany	Jul-2020	0.5	1	2	0.125	128	0.03	0.6	0.25	0.25
	Brittany	Aug-2020	1	0.5	1	0.125	**256**	0.03	0.6	0.125	0.25
F26	Brittany	Dec-2019	1	1	2	0.125	128	0.06	1.2	0.5	0.5
F27	New Aquitaine	Nov-2019	1	1	1	0.125	**>256**	**>8**	**>154**	**64**	4
F28	Brittany	Aug-2013	**>32**	1	2	0.125	**>256**	0.03	0.6	0.5	0.5
F29	Other area	Feb-2016	1	1	1	0.06	**>256**	0.25	4.8	**>64**	4
F30	Brittany	Jun-2019	1	2	2	0.25	**256**	0.06	1.2	0.5	0.5
F31	Brittany	Aug-2021	1	2	2	0.125	**256**	0.03	0.6	0.25	0.5
F32	Other area	Sep-2016	0.5	0.5	2	0.25	**>256**	0.125	2.4	0.25	0.5
F33	New Aquitaine	Jul-2013	2	4	4	0.5	64	0.06	1.2	0.25	0.5
	New Aquitaine	Nov-2014	2	**32**	**>32**	**2**	**>256**	1	19.25	**>64**	8
	New Aquitaine	Sep-2017	2	16	16	1	**>256**	0.25	4.8	**>64**	16
	New Aquitaine	Nov-2018	1	16	16	**2**	**>256**	0.25	4.8	**64**	8
F34	Other area	Jul-2020	1	1	1	0.125	**>256**	0.125	2.4	0.25	0.5
F35	Other area	Sep-2015	**32**	1	2	0.25	**>256**	**>8**	**>154**	32	2
F36	Brittany	Jun-2014	0.5	1	1	0.06	**>256**	0.125	2.4	32	4
	Brittany	Jul-2014	1	2	1	0.125	**>256**	0.06	1.2	16	4
	Brittany	Oct-2014	1	1	2	0.125	**>256**	0.25	4.8	**64**	8
	Brittany	Aug-2015	1	2	1	0.125	**>256**	0.125	2.4	**64**	8
	Brittany	Oct-2015	1	1	2	0.125	16	0.03	0.6	0.25	0.25
	Brittany	Aug-2018	1	1	1	0.125	**>256**	0.125	2.4	**64**	8
	Brittany	Dec-2018	1	1	2	0.125	**>256**	0.25	4.8	**>64**	8
	Brittany	Jul-2019	2	2	2	0.125	**>256**	0.125	2.4	**>64**	8
	Brittany	Sep-2021	2	2	2	0.125	**>256**	1	19.25	**64**	8
F37	Brittany	Sep-2014	**>32**	1	1	0.125	**>256**	**>8**	**>154**	32	2
F38	Brittany	Sep-2015	16	4	8	0.5	**>256**	4	77	**>64**	16
F39	Brittany	Nov-2013	1	2	2	0.125	**256**	0.06	1.2	0.25	0.25
	Brittany	Sep-2014	1	0.5	1	0.06	**256**	0.06	1.2	0.25	0.25
F40	New Aquitaine	Jul-2015	1	0.5	1	0.06	**>256**	0.125	2.4	**64**	8
F41	Brittany	Aug-2013	**>32**	4	16	1	**>256**	0.06	1.2	0.5	2
F42	Other area	Jul-2017	0.5	0.5	1	0.06	64	0.03	0.6	0.25	0.25
F43	Other area	Mar-2016	0.5	2	2	0.25	64	0.06	1.2	0.25	0.25
F44	Brittany	Apr-2019	0.5	1	2	0.125	128	0.03	0.6	0.25	0.25
F45	Brittany	Jan-2019	1	2	2	0.125	**256**	0.03	0.6	0.25	0.5
F46	Other area	Oct-2017	2	4	4	1	**>256**	**>8**	**>154**	0.5	0.5
F47	Other area	Mar-2019	1	16	**32**	1	**>256**	0.25	4.8	**>64**	8
F48	New Aquitaine	Jul-2012	2	2	2	0.25	**>256**	0.5	9.625	**>64**	8
	New Aquitaine	Sep-2012	2	**32**	16	1	**>256**	0.5	9.625	**64**	8
F49	Other area	Aug-2014	1	1	1	0.06	32	0.06	1.2	0.25	0.25
	Other area	Jul-2016	1	1	1	0.06	**256**	0.06	1.2	0.25	0.25
	Other area	Jul-2018	1	2	4	0.06	**>256**	**>8**	**>154**	32	4
	Other area	Jul-2021	1	1	2	0.125	**>256**	**>8**	**>154**	**64**	16
F50	Other area	Feb-2016	4	**32**	**>32**	**2**	**>256**	1	19.25	**>64**	16
	Other area	Mar-2016	2	8	8	**2**	**>256**	0.25	4.8	1	1
	Other area	Sep-2020	1	1	2	0.125	128	0.03	0.6	0.5	0.5
F51	Other area	Jul-2017	1	0.5	1	0.06	**>256**	0.125	2.4	**>64**	8
F52	Brittany	Jul-2020	1	1	1	0.125	**>256**	0.125	2.4	**>64**	4
	Brittany	Jul-2020	0.5	1	1	0.06	**>256**	0.125	2.4	**64**	8
	Brittany	Aug-2014	1	1	1	0.06	**256**	0.25	4.8	0.25	1

F53	Brittany	Jun-2016	1	16	8	1	>256	0.06	1.2	16	1
	Brittany	Aug-2016	0.5	0.5	1	0.125	128	0.06	1.2	0.125	0.125
F54	Other area	Jul-2020	0.5	4	2	0.125	256	0.06	1.2	0.25	0.5
F55	Brittany	Aug-2021	2	2	8	0.5	256	0.06	1.2	32	4
F56	Brittany	Aug-2015	4	32	32	2	>256	1	19.25	>64	16
	Brittany	Jul-2016	0.5	1	1	0.06	>256	0.125	2.4	>64	8
F57	Brittany	May-2019	0.5	1	2	0.25	>256	0.06	1.2	0.25	0.25
F58	Other area	Jun-2014	0.5	0.5	1	0.06	64	0.06	1.2	0.25	0.25
F59	New Aquitaine	Dec-2012	4	32	16	2	>256	1	19.25	>64	16
F59	New Aquitaine	Sep-2014	1	1	1	0.06	>256	0.06	1.2	>64	8
F60	Brittany	Jul-2019	1	1	2	0.125	256	0.06	1.2	0.5	0.5
F61	Other area	Jun-2015	16	>32	>32	>2	>256	>8	>154	>64	16
F62	Other area	Sep-2012	>32	1	1	0.125	>256	>8	>154	32	4
	Other area	Sep-2012	>32	8	8	0.5	>256	>8	>154	32	4
	Other area	Jul-2013	>32	0.5	0.5	0.06	>256	>8	>154	32	4
	Other area	Aug-2013	>32	2	1	0.125	>256	>8	>154	32	8
	Other area	Aug-2013	>32	1	2	0.06	>256	>8	>154	32	4
	Other area	Nov-2013	2	16	16	1	>256	0.25	4.8	64	4
	Other area	Jan-2014	1	0.5	1	0.06	>256	0.03	0.6	32	4
	Other area	Jul-2014	1	16	16	0.5	>256	0.25	4.8	>64	4
	Other area	Aug-2014	2	16	32	0.5	>256	0.25	4.8	64	2
	Other area	Sep-2014	>32	1	1	0.06	>256	>8	>154	64	2
	Other area	Oct-2014	>32	16	16	1	>256	>8	>154	>64	8
	Other area	Nov-2014	1	4	2	0.25	256	0.06	1.2	8	1
	Other area	Jun-2015	>32	16	16	1	>256	>8	>154	>64	16
	Other area	Aug-2015	0.5	1	1	0.125	128	0.06	1.2	0.25	0.25
	Other area	Aug-2015	>32	16	16	0.5	>256	>8	>154	>64	8
	Other area	Oct-2015	>32	16	32	2	>256	>8	>154	>64	16
	Other area	Dec-2015	2	16	16	1	>256	0.5	9.625	>64	8
	Other area	Sep-2016	1	1	1	0.125	256	0.03	0.6	0.25	0.5
	Other area	Sep-2016	1	16	16	0.5	>256	0.25	4.8	64	8
	Other area	Sep-2016	>32	8	16	0.5	>256	>8	>154	>64	8
	Other area	Oct-2016	>32	16	16	1	>256	>8	>154	64	16
	Other area	Nov-2016	>32	1	2	0.125	>256	>8	>154	64	8
	Other area	Mar-2017	>32	8	16	0.5	>256	>8	>154	64	8
	Other area	Aug-2017	>32	1	2	0.06	>256	>8	>154	>64	4
	Other area	Sep-2017	>32	8	8	0.5	>256	8	154	>64	4
	Other area	Sep-2017	>32	2	2	0.25	>256	>8	>154	64	4
	Other area	Oct-2017	>32	2	2	0.25	>256	>8	>154	64	8
	Other area	Dec-2017	1	16	16	1	>256	0.25	4.8	>64	8
	Other area	Oct-2018	>32	2	2	0.25	>256	>8	>154	>64	4
	Other area	Jul-2019	1	16	16	1	>256	0.25	4.8	>64	8
	Other area	Aug-2019	32	2	2	0.25	>256	>8	>154	64	4
	Other area	Sep-2019	>32	2	2	0.125	>256	>8	>154	64	8
	Other area	Oct-2019	>32	2	2	0.25	>256	>8	>154	>64	16
	Other area	Jun-2020	2	32	>32	2	>256	1	19.25	>64	32
	Other area	Jul-2020	8	>32	>32	>2	>256	1	19.25	>64	32
	Other area	Aug-2020	2	32	16	1	>256	1	19.25	>64	16
	Other area	Sep-2020	>32	2	2	0.125	>256	>8	>154	64	2
	Other area	Sep-2021	16	>32	>32	>2	>256	2	38.5	>64	>32
	Other area	Sep-2021	0.5	1	4	0.125	256	0.03	0.6	0.25	0.25
F63	Brittany	Jul-2020	1	2	2	0.125	>256	0.06	1.2	0.25	0.5
F64	Brittany	Aug-2017	1	0.5	1	0.06	>256	0.125	2.4	32	8
	Brittany	Aug-2018	>32	1	2	0.125	>256	>8	>154	64	4
F65	Brittany	Dec-2013	1	2	1	0.125	128	0.06	1.2	0.25	0.5
F66	Brittany	Sep-2019	1	1	2	0.125	256	0.06	1.2	0.5	0.5
	Brittany	Aug-2013	1	1	2	0.125	>256	0.06	1.2	1	0.5
	Brittany	Dec-2013	1	1	1	0.125	64	0.06	1.2	0.25	1
	Brittany	Jul-2014	1	1	2	0.125	128	0.03	0.6	0.25	0.25
	Brittany	Aug-2014	1	1	1	0.125	128	0.03	0.6	0.25	0.25
	Brittany	Aug-2014	0.5	0.5	1	0.06	128	0.03	0.6	0.25	0.25
	Brittany	Aug-2014	1	1	2	0.125	256	0.03	0.6	0.25	0.5
	Brittany	Aug-2014	>32	2	2	0.25	>256	>8	>154	0.25	0.5
	Brittany	Aug-2014	1	1	1	0.06	128	0.06	1.2	0.25	0.25
	Brittany	Sep-2014	1	8	0.5	0.125	32	0.125	2.4	0.25	0.25
	Brittany	May-2015	>32	1	1	0.06	>256	>8	>154	32	4
	Brittany	May-2015	1	1	1	0.125	64	0.03	0.6	<0.125	0.25
	Brittany	Jul-2015	1	1	0.5	0.06	128	0.06	1.2	0.25	0.25
	Brittany	Jul-2015	2	1	1	0.125	>256	0.125	2.4	32	2

F67	Brittany	Jul-2015	0.5	1	2	0.125	128	0.03	0.6	0.25	0.25
	Brittany	Aug-2015	1	1	1	0.25	128	0.03	0.6	0.5	0.25
	Brittany	Jul-2016	**>32**	4	8	1	**>256**	**>8**	**>154**	**>64**	16
	Brittany	Jul-2016	0.5	1	1	0.125	16	0.03	0.6	0.25	0.25
	Brittany	Aug-2016	1	1	1	0.125	64	0.03	0.6	0.25	0.25
	Brittany	Aug-2016	1	1	2	0.25	**256**	0.125	2.4	0.5	0.5
	Brittany	Apr-2017	**>32**	0.5	1	0.06	**>256**	**>8**	**>154**	32	4
	Brittany	May-2017	0.5	0.5	1	0.03	**>256**	0.06	1.2	0.25	0.25
	Brittany	Jul-2017	1	0.5	1	0.06	**256**	0.06	1.2	0.5	0.25
	Brittany	Aug-2017	4	0.5	1	0.125	64	0.06	1.2	0.5	0.5
	Brittany	Aug-2017	**>32**	1	2	0.125	**>256**	**>8**	**>154**	**64**	8
	Brittany	Oct-2017	1	2	2	0.125	128	0.06	1.2	0.25	0.5
	Brittany	Jan-2019	1	1	4	0.125	128	0.06	1.2	0.25	0.25
	Brittany	Jun-2019	0.5	0.5	1	0.06	**256**	0.06	1.2	0.25	0.25
	Brittany	Jul-2019	**>32**	1	2	0.06	**>256**	**>8**	**>154**	**64**	4
	Brittany	Jul-2019	0.5	8	2	0.06	32	0.03	0.6	0.25	0.25
	Brittany	Aug-2020	1	1	1	0.125	128	0.03	0.6	0.25	1
	Brittany	Aug-2020	2	2	4	0.25	**256**	0.06	1.2	0.25	1
	Brittany	Aug-2021	0.5	1	2	0.06	128	0.06	1.2	0.25	0.25
F68	Brittany	Nov-2013	0.5	4	2	0.25	**>256**	**>8**	**>154**	0.25	0.5
F69	Brittany	Jul-2013	1	1	1	0.125	**>256**	**>8**	**>154**	<0.125	0.25
	Brittany	Aug-2013	1	2	2	0.125	**>256**	**>8**	**>154**	0.25	0.5
	Brittany	Aug-2013	1	2	2	0.125	**>256**	**>8**	**>154**	0.25	0.5
	Brittany	Jul-2014	1	1	2	0.125	**>256**	**>8**	**>154**	0.5	0.25
	Brittany	Aug-2014	0.5	1	1	0.06	**>256**	**>8**	**>154**	0.25	0.125
	Brittany	Aug-2014	2	2	8	0.5	**>256**	**>8**	**>154**	0.25	1
	Brittany	Jul-2015	1	1	2	0.125	**>256**	0.5	9.625	**>64**	8
	Brittany	Jul-2016	0.5	2	1	0.125	128	0.125	2.4	1	0.5
	Brittany	Sep-2016	0.5	0.5	1	0.03	128	0.03	0.6	0.25	0.25
	Brittany	Sep-2016	1	1	2	0.125	**>256**	0.03	0.6	0.5	0.25
	Brittany	Sep-2016	0.5	1	2	0.06	**>256**	0.06	1.2	0.25	0.25
	Brittany	Sep-2016	0.5	0.5	1	0.06	**256**	0.03	0.6	0.25	0.25
	Brittany	Jun-2017	1	1	2	0.125	128	0.06	1.2	0.5	0.25
	Brittany	Jun-2017	1	0.5	2	0.06	**256**	0.03	0.6	0.25	0.25
	Brittany	Jul-2017	0.5	1	1	0.06	64	0.03	0.6	0.25	0.25
	Brittany	Jul-2017	1	1	2	0.125	64	0.06	1.2	0.5	0.5
	Brittany	Apr-2018	2	8	8	1	64	0.125	2.4	**>64**	8
	Brittany	Jul-2018	1	1	2	0.125	**256**	0.06	1.2	0.25	0.5
	Brittany	Aug-2018	2	8	8	1	64	0.125	2.4	**>64**	8
	Brittany	Sep-2018	1	1	2	0.125	128	0.03	0.6	0.5	1
	Brittany	Aug-2019	**>32**	1	1	0.125	**>256**	**>8**	**>154**	**64**	4
	Brittany	Aug-2019	1	1	2	0.125	**256**	0.06	1.2	0.5	0.5
	Brittany	Aug-2019	0.5	1	1	0.125	**>256**	0.03	0.6	0.25	0.25
	Brittany	Aug-2020	2	1	2	0.125	128	0.03	0.6	8	0.5
	Brittany	Aug-2020	1	1	2	0.125	**>256**	0.03	0.6	0.5	0.5
F70	Other area	Mar-2019	1	8	8	0.5	**>256**	0.06	1.2	**>64**	2
	Other area	Apr-2019	1	<0.06	<0.06	0.008	**256**	0.06	1.2	0.25	0.25
	Other area	Apr-2019	1	<0.06	<0.06	0.016	128	0.06	1.2	0.25	0.25
F71	Brittany	May-2020	**>32**	1	2	0.06	**>256**	**>8**	**>154**	32	4
F72	Brittany	Aug-2014	0.5	1	2	0.125	128	0.06	1.2	0.5	0.25
	Brittany	Aug-2015	0.5	1	1	0.125	128	0.06	1.2	0.25	0.25
	Brittany	Jul-2016	1	1	2	0.125	128	0.06	1.2	0.25	0.25
	Brittany	Sep-2016	**>32**	1	1	0.06	**>256**	**>8**	**>154**	32	4
	Brittany	Sep-2016	0.5	1	4	0.06	**256**	0.03	0.6	0.5	0.25
	Brittany	Jul-2017	1	0.5	1	0.06	128	0.06	1.2	0.5	0.25
	Brittany	Jul-2017	1	4	2	0.25	128	0.06	1.2	0.25	0.5
	Brittany	Aug-2017	2	**32**	**>32**	**2**	**>256**	0.5	9.625	**>64**	16
	Brittany	May-2018	1	1	2	0.125	**256**	0.06	1.2	0.5	0.25
	Brittany	Jul-2018	1	1	2	0.125	**256**	0.06	1.2	0.25	0.25
F73	Brittany	Aug-2014	0.5	2	2	0.125	64	0.125	2.4	0.25	0.25
	Brittany	Sep-2015	1	4	2	0.25	**256**	0.125	2.4	0.25	0.5
	Brittany	Sep-2015	**>32**	0.5	1	0.125	**>256**	**>8**	**>154**	**64**	4
	Brittany	Sep-2015	**>32**	2	1	0.125	**>256**	**>8**	**>154**	**>64**	16
	Brittany	Oct-2015	**>32**	1	1	0.125	**>256**	**>8**	**>154**	**>64**	8
	Brittany	Aug-2016	0.5	1	4	0.25	64	0.03	0.6	0.25	0.5
	Brittany	Sep-2016	**>32**	0.5	1	0.125	**>256**	**>8**	**>154**	**64**	2
	Brittany	Aug-2017	1	2	2	0.25	**256**	0.125	2.4	0.25	0.5
	Brittany	Sep-2017	**>32**	0.5	2	0.06	**>256**	**>8**	**>154**	32	4
	Brittany	Jul-2018	1	1	2	0.25	**256**	0.06	1.2	0.25	0.25

	Brittany	Sep-2018	**32**	1	1	0.125	**>256**	**>8**	**>154**	**64**	8
F74	Brittany	Aug-2015	0.5	1	1	0.125	**>256**	0.06	1.2	0.25	1
	Brittany	Aug-2016	**32**	2	1	0.125	128	0.03	0.6	0.25	0.25
	Brittany	Aug-2017	**>32**	0.5	1	0.06	**>256**	**>8**	**>154**	32	4
	Brittany	Aug-2017	**32**	1	2	0.06	**>256**	**>8**	**>154**	16	1
	Brittany	Aug-2017	1	0.5	1	0.06	128	0.125	2.4	0.5	1
	Brittany	Oct-2017	1	1	2	0.125	128	0.06	1.2	0.5	0.25
	Brittany	Oct-2017	1	1	2	0.125	128	0.06	1.2	0.5	0.5
	Brittany	Mar-2018	1	1	2	0.25	128	0.06	1.2	0.5	0.25
	Brittany	May-2018	**>32**	1	2	0.25	**>256**	**>8**	**>154**	**64**	4
	Brittany	Jun-2018	1	1	2	0.125	128	0.06	1.2	0.25	0.25
	Brittany	Jul-2018	2	2	4	0.25	**256**	0.06	1.2	0.25	0.5
	Brittany	Aug-2018	0.5	1	2	0.125	128	0.06	1.2	0.25	0.25
	Brittany	Sep-2018	1	1	2	0.125	128	0.06	1.2	0.25	1
	Brittany	Sep-2018	1	1	2	0.125	**256**	0.06	1.2	0.25	0.25
F75	Brittany	Sep-2017	**32**	1	2	0.125	**>256**	**>8**	**>154**	**64**	8
F76	Other area	27/11/2018	0.5	1	0.5	0.03	32	0.03	0.6	**64**	4
F77	Brittany	Aug-2014	1	1	2	0.125	**>256**	0.03	0.6	**>64**	4
	Brittany	Nov-2014	2	1	2	0.125	**>256**	0.06	1.2	**>64**	8
	Brittany	May-2015	1	1	2	0.125	**>256**	<0.016	<0.016	**>64**	8
	Brittany	Jun-2015	2	1	1	0.125	**>256**	0.06	1.2	**>64**	4
	Brittany	Jul-2015	2	**32**	**32**	**2**	**>256**	1	19.25	**64**	8
	Brittany	Jul-2015	0.5	0.5	0.5	0.06	**>256**	0.25	4.8	**>64**	2
	Brittany	Aug-2015	1	2	2	0.125	**>256**	0.125	2.4	**>64**	8
	Brittany	May-2016	4	**>32**	**>32**	**>2**	**>256**	1	19.25	**>64**	16
	Brittany	Jul-2016	1	1	1	0.125	**>256**	0.03	0.6	**>64**	8
	Brittany	Aug-2016	4	**32**	**32**	**>2**	**>256**	0.5	9.625	**64**	16
	Brittany	Aug-2016	**>32**	**>32**	**32**	**2**	**>256**	**>8**	**>154**	**>64**	**32**
	Brittany	Jul-2018	4	**32**	**32**	**2**	**>256**	2	38.5	**>64**	16
	Brittany	Aug-2018	**>32**	1	2	0.125	**>256**	**>8**	**>154**	**64**	4
	Brittany	Aug-2018	2	**32**	**>32**	**2**	**>256**	1	19.25	**>64**	8
	Brittany	Sep-2018	2	**32**	**32**	**2**	**>256**	1	19.25	**64**	8
	Brittany	Jul-2019	2	**32**	**32**	**2**	**>256**	2	38.5	**>64**	16
	Brittany	Aug-2021	4	4	16	1	**256**	0.125	2.4	0.5	2
F78	Brittany	Sep-2020	**>32**	1	2	0.25	**>256**	**>8**	**>154**	**64**	4
F79	Brittany	Nov-2015	1	1	2	0.125	**>256**	0.06	1.2	**>64**	8
	Brittany	Jul-2016	0.5	1	1	0.125	**>256**	0.125	2.4	**>64**	16
	Brittany	Sep-2016	1	1	2	0.06	**>256**	0.06	1.2	**>64**	8
	Brittany	Aug-2017	0.5	0.5	1	0.03	64	0.06	1.2	0.125	0.125
	Brittany	Sep-2017	2	2	2	0.125	128	0.06	1.2	0.25	0.5
	Brittany	Oct-2017	8	**>32**	**>32**	**>2**	**>256**	1	19.25	**>64**	**32**
	Brittany	Oct-2017	**>32**	2	1	0.125	**<256**	**>8**	**>154**	**64**	4
	Brittany	Oct-2017	4	**32**	**>32**	**2**	**>256**	1	19.25	**64**	16
	Brittany	Jun-2018	**>32**	1	2	0.25	**>256**	**>8**	**>154**	**64**	4
	Brittany	Jul-2018	**>32**	1	2	0.125	**>256**	**>8**	**>154**	**64**	4
	Brittany	Aug-2018	2	**32**	16	**2**	**>256**	0.5	9.625	**64**	16
F80	Brittany	Jun-2018	**>32**	2	4	0.25	**>256**	**>8**	**>154**	**64**	8
	Brittany	Jun-2018	2	**>32**	16	**>2**	**>256**	0.25	4.8	32	4
F81	Other area	Jul-2019	1	<0.06	0.25	0.008	**256**	0.06	1.2	0.25	0.5
F82	Brittany	Aug-2019	0.25	0.5	2	0.06	**256**	0.03	0.6	0.5	0.125
F83	New Aquitaine	May-2013	1	2	2	0.5	**>256**	0.125	2.4	0.125	0.25
	New Aquitaine	Feb-2014	1	2	1	0.125	**>256**	0.06	1.2	0.25	0.25
	New Aquitaine	Mar-2019	1	2	2	0.5	**>256**	0.125	2.4	0.25	0.25
	New Aquitaine	May-2021	1	4	2	0.25	**>256**	0.125	2.4	0.5	0.5
F84	New Aquitaine	Aug-2015	0.5	1	2	0.125	**256**	0.03	0.6	0.25	0.5
	New Aquitaine	Oct-2019	1	1	1	0.06	**>256**	0.25	4.8	**64**	8
	New Aquitaine	Jul-2021	1	1	1	0.06	**>256**	0.06	1.2	**64**	8
F85	New Aquitaine	Sep-2020	1	1	1	0.06	**>256**	0.25	4.8	**64**	8
F86	New Aquitaine	Jul-2016	2	1	2	0.25	**>256**	0.125	2.4	**>64**	8
	New Aquitaine	Apr-2017	1	0.5	2	0.125	**>256**	0.125	2.4	**64**	8
	New Aquitaine	Sep-2017	2	2	4	0.25	**>256**	0.25	4.8	**>64**	16
	New Aquitaine	Jan-2019	1	0.5	1	0.06	**>256**	0.25	4.8	**64**	8
F87	New Aquitaine	Dec-2015	0.5	1	1	0.06	**>256**	0.125	2.4	**64**	8
	New Aquitaine	Feb-2016	1	0.5	2	0.06	**>256**	0.125	2.4	**64**	8
	New Aquitaine	Aug-2017	1	1	2	0.125	**>256**	0.125	2.4	**>64**	8
	New Aquitaine	Sep-2018	1	1	1	0.06	**>256**	0.125	2.4	**64**	8
	New Aquitaine	Jul-2019	0.25	0.5	1	0.06	**>256**	0.03	0.6	32	2
	New Aquitaine	Jan-2020	0.5	0.5	1	0.03	**>256**	0.06	1.2	16	4
	New Aquitaine	Oct-2021	1	<0.06	<0.06	0.008	32	0.06	1.2	0.25	0.25

F88	New Aquitaine	Jan-2015	1	1	1	0.06	>256	0.125	2.4	64	8
	New Aquitaine	May-2015	1	0.5	1	0.125	>256	0.125	2.4	64	8
	New Aquitaine	Jul-2015	1	<0.06	0.125	0.008	32	0.03	0.6	0.25	0.25
	New Aquitaine	Jan-2016	1	1	1	0.125	>256	0.125	2.4	32	8
	New Aquitaine	Feb-2016	1	1	1	0.06	>256	0.125	2.4	64	8
	New Aquitaine	May-2017	1	1	1	0.125	>256	0.125	2.4	64	8
	New Aquitaine	Jul-2017	1	1	1	0.06	>256	0.06	1.2	64	8
	New Aquitaine	Jul-2017	1	0.5	2	0.125	>256	0.125	2.4	64	8
	New Aquitaine	Jul-2018	1	1	2	0.125	>256	0.125	2.4	>64	8
	New Aquitaine	Oct-2018	0.5	0.5	0.5	0.06	>256	0.125	2.4	64	4
	New Aquitaine	Apr-2019	1	0.5	1	0.06	>256	0.125	2.4	>64	4
	New Aquitaine	May-2019	1	1	0.5	0.06	>256	0.125	2.4	64	8
	New Aquitaine	Jun-2019	0.5	0.5	1	0.125	>256	0.06	1.2	32	8
	New Aquitaine	Oct-2019	1	1	0.5	0.03	>256	0.25	4.8	0.125	0.25
	New Aquitaine	Jun-2020	1	1	2	0.06	>256	0.25	4.8	>64	8
	New Aquitaine	Feb-2021	1	1	2	0.06	>256	0.06	1.2	32	4
	New Aquitaine	Jun-2021	0.5	0.5	1	0.03	>256	0.06	1.2	32	4
	New Aquitaine	Jul-2021	1	1	1	0.06	>256	0.25	4.8	32	8
	New Aquitaine	Jul-2021	1	0.5	0.5	0.03	>256	0.06	1.2	64	2
	New Aquitaine	Sep-2021	1	1	0.5	0.06	>256	0.25	4.8	64	8
	New Aquitaine	Oct-2021	0.125	2	1	0.125	>256	0.5	9.625	4	8
F89	New Aquitaine	Jul-2015	1	1	2	0.06	>256	0.06	1.2	32	8
	New Aquitaine	Jan-2016	0.5	0.5	1	0.06	>256	0.125	2.4	64	8
	New Aquitaine	Feb-2016	1	2	2	0.125	>256	0.125	2.4	32	8
	New Aquitaine	Sep-2016	1	1	2	0.06	>256	0.25	4.8	64	8
	New Aquitaine	Aug-2017	1	0.5	2	0.125	>256	0.06	1.2	64	8
	New Aquitaine	Jan-2018	1	0.5	1	0.06	>256	0.125	2.4	64	8
	New Aquitaine	Jul-2018	1	1	2	0.06	>256	0.125	2.4	64	4
	New Aquitaine	Jan-2019	1	1	2	0.125	>256	0.06	1.2	>64	8
	New Aquitaine	Jan-2020	4	1	2	0.125	>256	0.25	4.8	64	8
F90	New Aquitaine	Jul-2015	0.5	1	1	0.06	>256	0.06	1.2	64	8
F91	New Aquitaine	Jan-2015	1	0.5	1	0.06	>256	0.06	1.2	64	4
	New Aquitaine	Jan-2017	1	1	1	0.125	>256	0.125	2.4	64	4
	New Aquitaine	Feb-2017	1	1	2	0.06	>256	0.25	4.8	>64	4
	New Aquitaine	Jan-2019	1	1	1	0.06	>256	0.06	1.2	32	4
	New Aquitaine	Jul-2019	1	1	1	0.06	>256	0.25	4.8	64	8
	New Aquitaine	Sep-2020	1	1	1	0.06	>256	0.25	4.8	64	8
	New Aquitaine	Jul-2021	1	<0.06	<0.06	0.008	>256	0.03	0.6	0.25	0.25
F92	New Aquitaine	Nov-2015	1	1	1	0.06	>256	0.125	2.4	64	8
	New Aquitaine	Jul-2016	1	0.5	0.5	0.06	>256	0.03	0.6	0.25	0.5
	New Aquitaine	Feb-2017	1	1	1	0.125	>256	0.06	1.2	64	8
	New Aquitaine	Jan-2018	1	0.5	2	0.125	>256	0.125	2.4	64	4
	New Aquitaine	Jul-2018	1	0.5	1	0.06	>256	0.125	2.4	64	8
	New Aquitaine	Aug-2018	1	1	2	0.125	>256	0.125	2.4	64	8
	New Aquitaine	Sep-2019	1	1	2	0.125	>256	0.125	2.4	64	8
	New Aquitaine	May-2020	1	1	1	0.06	>256	0.06	1.2	32	8
	New Aquitaine	Aug-2021	1	1	2	0.125	>256	0.125	2.4	>64	8
F93	New Aquitaine	Jan-2015	2	1	1	0.125	>256	0.25	4.8	>64	8
	New Aquitaine	Sep-2015	1	1	1	0.125	>256	0.125	2.4	64	8
	New Aquitaine	Feb-2016	0.5	1	1	0.06	>256	0.06	1.2	32	4
	New Aquitaine	Sep-2016	1	0.5	1	0.125	>256	0.06	1.2	0.25	0.25
	New Aquitaine	Jul-2019	1	1	2	0.06	>256	0.25	4.8	>64	8
	New Aquitaine	Jul-2019	1	0.25	2	0.06	128	0.06	1.2	0.5	0.5
	New Aquitaine	Jul-2020	2	1	2	0.125	>256	0.25	4.8	>64	8
F94	New Aquitaine	Aug-2020	1	1	1	0.125	>256	0.25	4.8	>64	8
F95	New Aquitaine	Aug-2016	1	1	2	0.125	>256	0.125	2.4	64	8
	New Aquitaine	Jul-2019	1	<0.06	<0.06	0.008	64	0.03	0.6	0.25	0.25
	New Aquitaine	Jan-2020	1	1	1	0.06	>256	0.125	2.4	64	8
	New Aquitaine	Aug-2020	1	1	1	0.06	>256	0.125	2.4	64	8
F96	New Aquitaine	Sep-2016	1	1	2	0.125	64	0.06	1.2	0.5	0.5
	New Aquitaine	Jan-2017	1	1	2	0.06	>256	0.06	1.2	64	8
	New Aquitaine	Feb-2020	1	0.5	1	0.125	>256	0.06	1.2	16	2
	New Aquitaine	Feb-2020	1	0.5	0.5	0.125	>256	0.25	4.8	64	8
